# Combination of Imipenem-Cilastatin-Relebactam and Amoxicillin in the Antibiotic Regimen in Two Cases of Mycobacterium abscessus Lung Infection

**DOI:** 10.7759/cureus.65112

**Published:** 2024-07-22

**Authors:** Anastasios I Vogiatzoglou, Maria Hadji Μitrova, Eleni Papadaki, Maria Sionidou, Anna Nikopoulou, Fanοurios Kontos, Dimitrios Papaventsis, Apostolos Papavasileiou, Katerina Manika

**Affiliations:** 1 Pulmonology and Tuberculosis Department, General Hospital of Thessaloniki “Georgios Papanikolaou”, Aristotle University of Thessaloniki, Thessaloniki, GRC; 2 Internal Medicine Department, General Hospital of Thessaloniki “Georgios Papanikolaou”, Thessaloniki, GRC; 3 Laboratory of Clinical Microbiology, University General Hospital of Athens “Attikon”, Athens, GRC; 4 Microbiology, National Reference Laboratory for Mycobacteria, General Hospital of Thoracic Diseases of Athens “Sotiria”, Athens, GRC; 5 Antituberculosis Department - Multidrug-Resistant Tuberculosis Unit, General Hospital of Thoracic Diseases of Athens “Sotiria”, Athens, GRC

**Keywords:** nontuberculous mycobacteria (ntm), amoxicillin, imipenem/cilastatin/relebactam, relebactam, mycobacterium abscessus

## Abstract

*Mycobacterium abscessus* is a difficult-to-treat, multidrug-resistant human pathogen. Relebactam has been shown to inhibit *M. abscessus* β-lactamase (BLA_Mab_) and increase the activity of imipenem and amoxicillin. We present two cases of lung infection due to *M. abscessus*, one caused by *M. abscessus*subsp*. massiliense *and the other by subsp*. abscessus*. Both strains showed moderate sensitivity to imipenem, and the second strain was also resistant to macrolides. A multidrug antibiotic regimen was administered in both cases, which included imipenem/cilastatin/relebactam adjusted to the estimated glomerular filtration rate (eGFR) and amoxicillin for three months. The regimen was well tolerated and both patients improved both clinically and radiologically after the first phase of treatment. The results of our patients indicate that the combination of imipenem/cilastatin/relebactam and amoxicillin could be used in the future in difficult infections by *M. abscessus*.

## Introduction

*Mycobacterium abscessus* is an increasingly prevalent human pathogen, which is considered a global health threat [1,2], representing 3-13% of all nontuberculous mycobacteria (NTM) pulmonary diseases [3]. It is the most common etiological agent of lung disease caused by rapid-growing NTM [3] and the second most frequent NTM pulmonary disease pathogen [4]. Its transmission is mainly related to environmental factors, such as soil and water resources [5]; however, transmission in healthcare settings between patients with cystic fibrosis has been reported [6,7]. *M. abscessus* can cause severe lung disease, either upper lobe fibrocavitary or nodular form [3], or disseminated disease related to immunosuppression [4], as well as skin or soft tissue infections associated with invasive procedures [1,2,4].

Diagnosis of *M. abscessus*, like other NTM infections, requires a combination of clinical and radiographic findings and microbiological confirmation [8]. The microbiological methods used are smear microscopy, culture, and molecular methods [8,9]. Culture remains the gold standard and is required to identify NTM at the subspecies level [9-11]. For pulmonary disease, positive culture is required to diagnose NTM infection on at least two separate sputum specimens or a single lower respiratory specimen [10,11]. Molecular methods, such as line probe hybridization, polymerase chain reaction (PCR) methods, and DNA sequencing, have replaced older tests for NTM identification and can be performed at the subspecies level [8,9]. GenoType NTM-DR (NTM-DR) is an example that combines PCR and reverse hybridization [12].

*M. abscessus* is difficult to treat [10] with a low cure rate of 30-50% [3], due to its natural resistance to frontline agents used against tuberculosis (TB) and many other common antibiotics [1,13]. Treatment has two phases: the initial phase includes a combination of intravenous agents, such as imipenem, while the continuation phase includes oral and inhaled antibiotics. The optimal treatment duration is currently unknown [10].

*M. abscessus* is a multidrug-resistant pathogen [3]. Its endogenous class A β-lactamase (BLA_Mab_) causes resistance to most β-lactam antibiotics [1-3,13-19]. Relebactam, a newer β-lactamase inhibitor, seems to increase the in vitro activity of imipenem against *M. abscessus* [1,2,14-19] and to exhibit in vitro synergistic effect with imipenem and amoxicillin [1,14,15].

We present the cases of two patients with *M. abscessus* pulmonary infection who were treated with antibiotic combinations including relebactam together with imipenem and amoxicillin for three months. In both cases, moderate sensitivity to imipenem was observed.

## Case presentation

Patient 1

**Figure 1:**
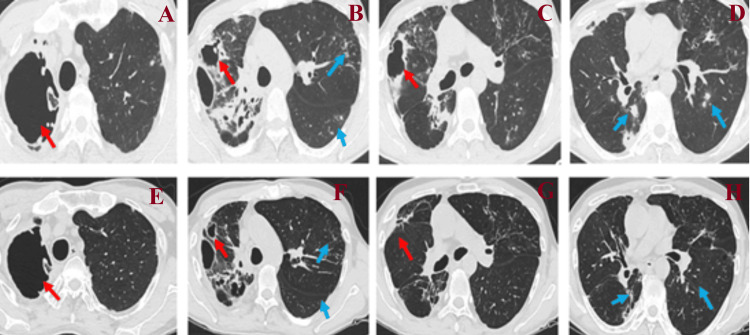
CT scan of patient 1 Figure panels A, B, C, and D present imaging before treatment, and panels E, F, G, and H present imaging at the end of the initial phase of treatment. A decrease in the size of the large cavities (red arrows) and a decrease in the extent of pulmonary infiltrates (tree-in-bud lesions) (blue arrows) are observed.

Patient 1 was a 52-year-old man from Georgia, who had been treated for pulmonary TB 25 years ago. He presented with cough, morning sputum, weakness, and weight loss during the last three years, despite taking a second course of anti-TB treatment. Chest CT scan revealed a large cavity in the right upper lobe, bronchiectasis in all lobes, and "tree in bud" lesions bilaterally (Figure 1). Microbiological examination of two sputum samples identified *M. abscessus subsp. massiliense*, using the NTM-DR (Bruker, United States) molecular method on the cultured material. Ziehl-Neelsen staining was negative. The drug susceptibility test presenting good sensitivity to macrolides and moderate sensitivity to imipenem is shown in Table 1.

**Table 1:** Drug sensitivity test

	Patient 1		Patient2	
Antibiotic agent	MIC (mg/mL)	Sensitivity	MIC (mg/mL)	Sensitivity
Amikacin	8	Sensitive	8	Sensitive
Cefoxitin	64	Moderately sensitive	32	Moderately sensitive
Ciprofloxacin	>4	Resistant	2	Moderately sensitive
Clarithromycin	0.12	Sensitive	>16	Resistant
Doxycycline	>16	Resistant	>16	Resistant
Linezolid	<1	Sensitive	8	Sensitive
Imipenem	16	Moderately sensitive	8	Moderately sensitive
Moxifloxacin	4	Resistant	2	Moderately sensitive
Trimethoprime/sulfamethoxazole	2/38	Sensitive	8/152	Resistant

The initial phase of treatment included a combination of intravenous imipenem/cilastatin/relebactam with oral amoxicillin, together with three other agents (Table 2). At the end of the three-month initial phase, the patient improved clinically, with the elimination of cough and sputum and an increase in body weight. The only adverse effect observed was mild nausea attributed to tigecycline. A new CT scan showed imaging improvement (Figure 1). The patient is currently in the seventh month of the continuation phase, which includes oral clofazimine, cotrimoxazole, azithromycin, and linezolid, as well as inhaled amikacin, showing a favorable response.

**Table 2:** First phase’s antibiotic regiment *Imipenem, cilastatin, and relebactam were administered in the pre-formulation form. The dose was adjusted to eGFR according to instructions in the Drug’s Summary of Product Characteristics (SPC).

	Patient 1	Patient 2
Antibiotic	Administration - dose	Administration - dose
imipenem*	iv 500 mg q.i.d.	iv 400 mg q.i.d.
cilastatin*	iv 500 mg q.i.d.	iv 400 mg q.i.d.
relebactam*	iv 250 mg q.i.d.	iv 200 mg q.i.d.
amoxicillin	pos 1000 mg t.i.d.	pos 1000 mg t.i.d.
amikacin	iv 15 mg/kg – five times a week	iv 15 mg/kg – five times a week
tigecycline	iv 50 mg q.d.	50 mg q.d.
azithromycin	pos 500 mg q.d.	-
linezolid	-	pos 600 mg q.d.
clofazimine	-	pos 100 mg q.d.

Patient 2

**Figure 2:**
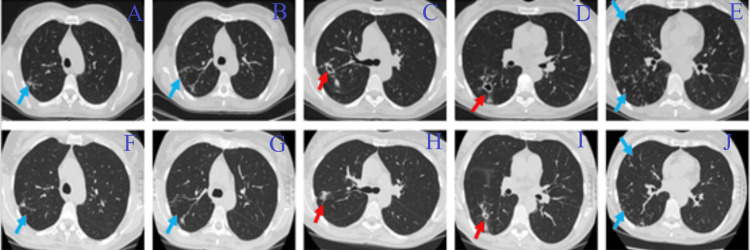
CT scan of patient 2 Figure panels A, B, C, D, and E present imaging before treatment, and panels F, G, H, I, and J present imaging at the end of the initial phase of treatment. A decrease in the size of the cavities (red arrows) and a decrease in the extent of tree-in-bud lesions (blue arrows) are observed.

The second case was a 54-year-old Greek woman, with untreated psoriasis and sclerosing cholangitis. She presented with hemoptysis and paroxysmal cough during the past seven years. Chest CT scan revealed bilateral "tree-in-bud" lesions, ground-glass lesions bilaterally, a small cavity in the right lower lobe, and calcified granulomas in both lungs (Figure 2). *M. abscessus *subsp.* abscessus* was microbiologically isolated from two sputum samples, using the NTM-DR (Bruker) molecular method on the cultured material. Ziehl-Neelsen staining was negative. The drug susceptibility test showing resistance to macrolides and moderate sensitivity to imipenem is presented in Table 1.

The initial phase of treatment included a combination of intravenous imipenem/cilastatin/relebactam with oral amoxicillin, together with four other antibiotics (Table 2). At the end of the initial phase, the patient significantly improved, with complete remission of cough and no further hemoptysis. She presented some adverse effects: persisting nausea attributed to tigecycline, mild and non-significant numbness of the legs, and a mild reversible drop in white blood cells attributed to linezolid. A new chest CT scan also showed improvement (Figure 2). Patient 2 is currently in the fifth month of the continuation phase, under oral clofazimine, linezolid at a reduced dose, bedaquiline, and moxifloxacin, as well as inhaled amikacin, with favorable response.

## Discussion

In the cases presented here, *M. abscessus *pulmonary disease was treated with a combination of imipenem/cilastatin/relebactam and amoxicillin for three months. Both patients tolerated the treatment well and showed a favorable response clinically and radiologically.

A major mechanism of resistance of *M. abscessus* is the expression of an endogenous class A β-lactamase, BLA_Mab_, which hydrolyses β-lactam agents and makes them ineffective [1-3,14]. Only imipenem and cefoxitin maintain some activity because they are slowly hydrolyzed by BLA_Mab_ [20,21]. This enzyme is not inhibited by older β-lactamase inhibitors, such as clavulanic acid, sulbactam, and tazobactam [15].

The role of newer β-lactamase inhibitors in the treatment of *M. abscessus* has been recently investigated [1] and combinations of imipenem/avibactam and imipenem/relebactam have shown increased bactericidal activity [2]. However, avibactam is only available in pre-formulation with ceftazidime, which increases the antibiotic burden on patients. Therefore, the combination of imipenem with relebactam is considered a future frontline option [2].

Relebactam has been shown to reduce the MIC of imipenem against *M. abscessus* isolates [15-19]. Fröberg et al. have found this reduction to be about 50% [19], while Misawa et al. support that relebactam lowers the MIC of β-lactams against all *M. abscessus* subspecies [17]. MIC reduction is attributed to the effective inhibition of BLA_Mab_ by relebactam [1,2,14-19]. Recent data also show a synergistic activity between imipenem, relebactam, and amoxicillin, which makes *M. abscessus* susceptible to amoxicillin [1,14,15]. In addition, the combination of more than two β-lactams (e.g., imipenem and amoxicillin) achieves better inhibition of D,D-transpeptidases, which are essential for mycobacterium’s cell wall, leading to its destruction [15]. Dousa et al. suggest that the dual β-lactam combination has a stronger anti-*abscessus *effect in vitro than the addition of relebactam to a single β-lactam [22]. Le Run et al. support that the effect of relebactam is probably greater in infected macrophages than in vitro because BLA_Mab _ is produced to a greater extent by intracellular bacteria than by bacteria growing in the culture [14].

However, these effects have not been adequately studied in the clinical setting. Beech et al. treated a patient with a soft tissue infection due to *M. abscessus* with an antibiotic combination containing imipenem/cilastatin/relebactam, but not amoxicillin, with good results [23]. Imipenem/cilastatin/relebactam was administered twice a day as a supplement to imipenem/cilastatin twice a day for twelve weeks [23]. In contrast to this regimen, we administered imipenem/cilastatin/relebactam four times a day for 12 weeks at a dose adjusted to the patients' eGFR, according to the drug’s Summary of Product Characteristics. On the other hand, data on the effectiveness of this combination in pulmonary disease are lacking.

## Conclusions

The two cases presented were treated with imipenem/cilastatin/relebactam and amoxicillin as part of the initial phase regimen for pulmonary infections caused by *M. abscessus* subsp. *massilience *and subsp. *abscessus*. No adverse effect related to relebactam or β-lactams was observed. Both patients showed clinical and imaging improvement after three months of treatment, suggesting that the combination of imipenem/cilastatin/relebactam and amoxicillin is safe and effective and may be an option for pulmonary infections due to *M. abscessus* in the future.
